# Effectiveness of physical activity promotion and exercise referral in primary care: protocol for a systematic review and meta-analysis of randomised controlled trials

**DOI:** 10.1186/s13643-019-1198-y

**Published:** 2019-12-05

**Authors:** Jean-Pierre Laake, Joanna Fleming

**Affiliations:** Unit of Academic Primary Care, Warwick Medical School, University of Warwick, Coventry, UK

**Keywords:** Physical activity, Exercise, Promotion, Referral, Primary care, Intervention, Randomised controlled trial, Systematic review, Meta-analysis

## Abstract

**Background:**

Physical inactivity is the fourth leading risk factor for global mortality. Reducing sedentary behaviour and increasing physical activity are efficacious for improving many physical and mental health conditions including cardiovascular disease, type 2 diabetes and depression. Reducing sedentary behaviour and increasing physical activity can also be effective at reducing obesity; however, sedentary behaviour and reduced physical activity are also associated with mortality independently. Despite this, most adults in the UK do not currently meet the UK Chief Medical Officers’ guidelines for weekly physical activity. As most adults visit their general practitioner at least once a year, the primary care consultation provides a unique opportunity to deliver exercise referral or physical activity promotion interventions. This is a protocol for a systematic review of randomised controlled trials for the effectiveness of physical activity promotion and referral in primary care.

**Methods:**

A comprehensive literature search of Embase, MEDLINE (Ovid), Web of Science (Core Collection), Scopus, CINAHL, PsycINFO, and The Cochrane Library (CENTRAL) will be conducted for studies with a minimum follow-up of 12 months that report physical activity as an outcome measure (by either self-report or objective measures) including an intention to treat analysis. The authors will screen papers, first by title and abstract and then by full text, independently assess studies for inclusion, appraise risk of bias and extract data. The quality of the evidence will be assessed using the GRADE (Grading of Recommendations Assessment, Development and Evaluations) approach. The primary outcome will be participation in physical activity at 12 months. Pooled effects will be calculated using random effects models. Results will be submitted for publication in a peer-reviewed journal and for presentation at UK national primary care conferences.

**Discussion:**

This systematic review and meta-analyses will summarise the evidence for the effectiveness of physical activity promotion and referral as interventions for improving physical activity, as well as whether studies using objective measures of physical activity have similar effects to those studies using self-report measures. This knowledge has importance for primary care clinicians, patients and, given the focus of the recent NHS long-term plan on preventive medicine, those making policy decisions.

**Systematic review registration:**

The protocol is registered with PROSPERO the international prospective register of systematic reviews, ID CRD42019130831

## Background

Physical inactivity is the fourth leading risk factor for global mortality and contributes towards four out of five of the remaining top six leading risk factors for global mortality [1]. Regular physical activity helps in the management and prevention of common chronic conditions including, depression, anxiety, obesity (adiposity), cerebrovascular accident, cancers, type 2 diabetes, and musculoskeletal conditions [2–4]. Due to the recognised relationship between increased physical activity and reduced morbidity and mortality, the UK Chief Medical Officers have created guidelines for minimum levels of physical activity. Firstly, adults should be physically active for a minimum of either 2.5 h at moderate intensity or 1.25 h at vigorous intensity per week (or a combination of both) in bouts of ≥ 10 min. Additionally, adults should exercise to improve muscle strength on at least two days per week and should avoid time spent sedentary [4]. Despite this, in England, the number of adults meeting the UK guidelines for physical activity has not significantly increased since 2012. Only 66% of men and 58% of women met the aerobic guidelines in 2016 and just 31% of men and 23% of women met both the aerobic and muscle-strengthening guidelines according to the National Health Service (NHS) Health Survey for England [5].

The primary care consultation provides a unique opportunity for the prevention of disease [6]. In the UK, most adults visit their general practitioner (GP) at least once a year [7] and patients are open to being supported by their primary care physician to adopt preventive lifestyle adaptations [8]. Within primary care physical activity can be promoted in different ways, namely physical activity promotion (brief verbal advice/encouragement, written materials) and exercise referral (referral to exercise programme, social prescribing). There has been a sustained increase in physical activity promotion and exercise referral schemes across the UK over the past three decades including GP referrals for gym or exercise classes (e.g. Fitter Futures in Warwickshire [9] or the Wales National Exercise Referral Scheme [10]). More recently the parkrun practice initiative, a collaboration between the Royal College of General Practitioners (RCGP) and parkrun, was launched in 2018 [11]. Recent evidence suggests that even achieving small increases in physical activity can lead to significant health benefits [12] and there is evidence that exercise promotion in primary care may be effective; however, this evidence is almost exclusively from meta-analyses of trials that have used self-report outcome measures [13–15].

Other primary preventive interventions, including brief and very brief interventions to reduce alcohol consumption and promote smoking cessation in primary care, have been shown to be effective [16–18]. This review will seek to update previous reviews of trials of interventions for physical activity in primary care. These have been limited by the inclusion of trials with short follow-up duration and the paucity of randomised controlled trials for exercise referral [14, 15, 19]. Furthermore, these previous reviews have been unable to carry out subgroup analysis according to whether studies used self-report or objective outcome measures due to the paucity of studies of exercise promotion or referral using objective measures of physical activity [14, 15]. While subsequent systematic reviews have been published, there have been further trials of physical activity promotion in primary care which have used objective outcome measures [20–23]. As such, the current systematic review will seek to update the available evidence.

This is a protocol for a systematic review of randomised controlled trials of primary care-based physical activity promotion and referral interventions for adults with at least 12 months of follow-up. There are two main objectives for which meta-analyses will be carried out. The first is to determine whether there are sustained effects of exercise referral on physical activity participation at 12-month follow-up (as have previously been shown for exercise promotion [15]). The second is to compare whether results from studies using objective outcome measures of physical activity differ from those using self-report measures. Assessment for heterogeneity will be achieved through meta-regression and subgroup analysis.

In addition to including new trials, the current systematic review differs from those previously published as it will only include studies with self-report or objective measures of physical activity as an outcome and will use meta-regression and subgroup analysis to explore potential heterogeneity of summary effects. The results of this analysis will benefit primary care clinicians, patients, commissioners and those making policy decisions and this is particularly pertinent in the UK given the focus of the recent NHS long-term plan on preventive medicine [24].

## Methods/design

The objective of this article is to describe the protocol for this systematic review and meta-analyses according to the preferred reporting items for systematic review and meta-analysis protocols (PRISMA-P) [25]. The protocol is registered with PROSPERO the international prospective register of systematic reviews, ID CRD42019130831 [26].

### Eligibility criteria

Studies will be selected according to the criteria described as follows.

#### Types of studies

Only randomised controlled trials will be included. These are defined as trials where allocation to the intervention and comparison group was described as randomised at the participant level or at the cluster level and which include an intention to treat analysis.

#### Types of participants

Only studies of adults and adolescents aged ≥ 16 years of age will be included. There will be no restrictions based on participants’ medical histories or medical conditions.

#### Types of interventions

Studies including interventions consisting of physical activity promotion (brief verbal advice/encouragement, written materials) and/or exercise referral (referral to exercise programme, social prescribing) will be included.

#### Comparators

The treatment in the comparator arm must be less intensive than the treatment in the intervention arm, e.g. depending on the treatment arm comparative arm(s) can be usual care, physical activity promotion or exercise referral or non-physical activity/exercise interventions.

#### Outcome

Participation in physical activity per unit time. This may be all physical activity or physical activity at a specified intensity, measured using either self-report measures, e.g. International Physical Activity Questionnaires (IPAQ) or objective measures, e.g. accelerometers, pedometers [27, 28]. There will be no restrictions based on the type of self-report or objective measures used.

#### Follow-up times

Only studies with follow-up data at ≥ 12 months post randomisation will be included.

#### Setting

Only studies where the intervention/referral was in the primary care setting will be included. There will be no restrictions based on the type of primary care setting.

### Search strategies

A comprehensive search for relevant randomised controlled trials will be performed between October and November 2019 in the following electronic bibliographic databases (from inception onwards): MEDLINE (Ovid), Embase, Web of Science (Core Collection), Scopus, CINAHL, PsycINFO, and The Cochrane Library (CENTRAL). A draft search methodology for MEDLINE and Web of Science (Core Collection) is available online (Additional file 1). A research librarian with expertise in search strategies for systematic reviews will further help develop the searches based on the following domains using Medical Subject Headings (MeSH) where possible “exercise/physical activity”, “primary care/general practice/family practice”, “randomised controlled trial”. Reference lists of previous reviews and included papers will be searched to check for any further trials. The flow of results generated and reasons for exclusion will be presented in the PRISMA flow chart [29].

### Data collection and analysis

Relevant records will be screened through a two-stage process. In the first stage, titles and abstracts will be screened by two investigators. Studies considered not to be relevant (according to the eligibility criteria listed above) will be excluded. In the second stage, two investigators will examine the remaining full text reports for concordance with the inclusion criteria.

### Data extraction and management

Two authors (JPL and JF) will independently extract the data using a structured form. Any discrepancies in the data extraction or inclusion/exclusion of trials will be resolved by referring to the original papers. The authors will not be blinded to article results, authors, or institutions. In addition to outcomes, data extraction will include information pertaining to authors, year of publication, country of origin, number of participants, participant demographics (age, sex, measures or proxy measures of adiposity, health status), intervention type (exercise intervention versus physical activity promotion), intervention frequency, intervention duration, setting of intervention/referral (clinical versus non-clinical), comparator group, outcome measure, outcome measurement method (self-report versus objective). The results will be presented in the form of a narrative synthesis summarising results by intervention type alongside a detailed summary of findings table.

### Risk of bias and quality of evidence

Risk of bias will be assessed using the Cochrane risk-of-bias tool (RoB 2), and as recommended by the *Cochrane Handbook for Systematic Reviews of Interventions* [30], the following domains of bias will be reported for each study: (i) bias arising from the randomisation process (selection bias), (ii) bias due to deviations from intended interventions (performance bias), (iii) bias due to missing outcome data (attrition bias), (iv) bias in the measurement of the outcome (detection bias/response bias), (v) bias in the selection of the reported result (reporting bias) [31]. Each domain will be judged as low, some concerns or high with reasoning discussed. Two authors (JPL and JF) will independently assess each study and disagreements will be resolved by referring to the original papers. The results will be presented in a summary of findings table according to the GRADE (Grading of Recommendations Assessment, Development and Evaluations) approach as high, moderate, low or very low [32].

### Data synthesis

So that studies using both dichotomous and continuous outcome data can be included in the meta-analyses, (relative risk) odds ratios with 95% confidence intervals will be calculated for studies with dichotomous data and standardised mean differences with 95% confidence intervals will be calculated for studies with continuous outcome data. Separate meta-analyses will be carried out for studies where incomparable outcome measures are used. For example, studies reporting dichotomous data on reaching physical activity targets or not will be included in a separate meta-analysis to those reporting total minutes of physical activity as a continuous variable or frequency of episodes of physical activity (a proxy measure of total duration in minutes) as a continuous variable. If studies report both, then they will be included in both meta-analyses. Pooled effects will be calculated using a random effects model from the closest follow-up measurement ≥ 12 months of follow-up. The heterogeneity of results will be quantified by the I^2^ statistic. Should there be missing data we will attempt to contact the original authors to obtain this. Any missing data collected or that we are unable to collect will be recorded.

### Subgroup analysis

Subgroup analyses and meta-regression will investigate (i) the effects of exercise referral versus physical activity promotion, (ii) the effects of outcome measure (i.e. self-report versus objective measure of physical activity). The heterogeneity of results will be quantified by the I^2^ statistic and will be explored by the analysis of subgroups as described above. It is expected that there will be less trials including objective measures of physical activity. However, if there are a sufficient number of trials included we will also do a subgroup analysis to investigate the effects of exercise referral versus physical activity promotion stratified by outcome measure. Pooled effects will be calculated using a random effects model. Publication bias will be assessed by visual inspection of a funnel plot and Egger’s test. The analyses will be performed according to the *Cochrane Handbook for Systematic Reviews of Interventions* [30].

## Discussion

Physical inactivity is a leading cause of premature deaths both in the UK and worldwide and is a modifiable risk factor for the development of a large number of metabolic-related chronic conditions such as cardiovascular disease, type 2 diabetes and osteoporosis [1, 3]. Furthermore, increasing physical activity can improve outcomes in patients with chronic disease and is effective at improving symptoms in patients with a range of neurological conditions such as depression, anxiety and chronic pain [2–4]. Offering physical activity promotion and exercise referral in the primary care setting provides an opportunity to support patients in making lifestyle changes to increase their physical activity, reduce their chances of premature death and improve their quality of life.

In this systematic review, we expect that we will be able to identify whether current evidence demonstrates exercise referral and physical activity promotion interventions are effective at increasing physical activity after 12 months. We hope that with the inclusion of more recent studies that concerns discussed in previous reviews of such interventions in primary care can be addressed, in particular those surrounding paucity of evidence and reliability of self-report outcome measures.

Any important protocol amendments will be recorded on PROSPERO. An abstract of the completed synthesis will be submitted for presentation at relevant national primary care conferences in the UK. The full manuscript will be submitted to a relevant peer-reviewed journal for consideration for publication. The results of this systematic review and meta-analyses will support patients, clinicians, health-care providers and decision makers by summarising the evidence base for different types of primary care interventions for increasing physical activity. This is particularly relevant in the UK given the focus of the recent NHS long-term plan on preventive medicine and the recent investment in social prescribing.

## Supplementary information

**Additional file 1.** Search V0.3.2.docx – MEDLINE & Web of Science Draft Search.

## Data Availability

Data sharing is not applicable to this article as no datasets were generated or analysed during the current study.

## Abbreviations

GRADE: Grading of Recommendations Assessment, Development and Evaluations
GP: General practitioner
IPAQ: International Physical Activity Questionnaires
MeSH: Medical Subject Headings
MBChB: Bachelor of Medicine, Bachelor of Surgery
NHS: National Health Service
PRISMA: Preferred Reporting Items for Systematic Review and Meta-Analysis
PRISMA-P: Preferred Reporting Items for Systematic Review and Meta-Analysis Protocols
RCGP: Royal College of General Practitioners
RoB2: Cochrane risk-of-bias tool
