# Supplementary material for: Effectiveness of physical activity promotion and exercise referral in primary care: protocol for a systematic review and meta-analysis of randomised controlled trials
Source: Syst Rev. 2019 Dec 5;8:303. doi: 10.1186/s13643-019-1198-y (PMC6894292; doi:10.1186/s13643-019-1198-y)
Supplement: Supplementary file 1 — Additional file 1. Search V0.3.2.docx – MEDLINE & Web of Science Draft Search. [file 13643_2019_1198_MOESM1_ESM.docx]

**Supplementary file**

**Draft for the search strategy for MEDLINE (Ovid)**

1 exp Exercise/ (184148)

2 exercise.mp. (301109)

3 exp Exercise Therapy/ (47683)

4 physical activity.mp. (85072)

5 1 or 2 or 3 or 4 (392924)

6 exp Primary Health Care/ (151108)

7 primary care.mp. (95405)

8 primary health care.mp. (84106)

9 exp Family Practice/ (64754)

10 family practice.mp. (66990)

11 exp General Practice/ (73882)

12 general practice.mp. (43834)

13 6 or 7 or 8 or 9 or 10 or 11 or 12 (283759)

14 exp Clinical Trial/ (840057)

15 5 and 13 and 14 (1359)

16 limit 15 to english language (1324)

**Draft for the search strategy for Web of Science (Core Collection)**

((TS=Exercise OR TS=(“Physical Activity”) OR TS=Run* OR TS=Swim* OR TS=Walk* OR TS=Jog* OR TS=(Stair AND Climb*) OR TS=(Resistance AND Train*) OR TS=(Endurance AND Train*) OR TS=(Interval AND Train*))

AND

(TS=(“Family Practice”) OR TS=(”General Practice”) OR TS=(“Primary Health Care”) OR TS=(“Primary Care”) OR WC=(Primary Health Care))

AND

(TS=(“Clinical Trial*”) OR TS=(“Controlled Trial*”) OR TS=(“Randomi?ed Trial*”) OR TS=RCT))

AND

LANGUAGE: (English)

(3725 Results)
